# Association of eight anthropometric indexes related to obesity with the prevalence of clinical osteoarthritis among American adults: a national cross-sectional study

**DOI:** 10.1186/s40001-025-03131-z

**Published:** 2025-09-26

**Authors:** Jingtao Huang, Xuan Zhang, Haoxian Tang, Shicheng Jia, Jiayou Chen, Rongji Liang, Qinglong Yang, Hanyuan Lin, Nan Luo, Yuxiang Ren, Jianjing Lin, Xintao Zhang

**Affiliations:** 1 Department of Sports Medicine and Rehabilitation, Peking University Shenzhen Hospital, Shenzhen, 518036 People’s Republic of China; 2 Shantou University Medical College, Guangdong Shantou, 515041 People’s Republic of China; 3 Department of Bone and Joint Surgery, Peking University Shenzhen Hospital, Shenzhen, Guangdong People’s Republic of China; 4 Department of Cardiology, The First Affiliated Hospital of Shantou University Medical College, Shantou, 515041 People’s Republic of China; 5 Department of Urology, The Second Affiliated Hospital of Shantou University Medical College, Shantou, , Guangdong People’s Republic of China; 6 Department of Psychiatry, Shantou University Mental Health Center, Shantou, Guangdong People’s Republic of China

**Keywords:** Anthropometric indexes, Osteoarthritis, Obesity, National health, Nutrition examination survey

## Abstract

**Objective:**

The aim of the current study is to investigate the association between clinical osteoarthritis (OA) and eight anthropometric indexes related to obesity, including non-hematological indexes (body mass index [BMI], body roundness index [BRI], weight-adjusted waist index [WWI], and waist-height ratio [WHtR]), and hematological indexes (triglyceride-glucose index [TyG], lipid accumulation product [LAP], visceral adiposity index [VAI], and waist triglyceride index [WTI]).

**Methods:**

Utilizing data from the National Health and Nutrition Examination Surveys (NHANES) spanning the years 2005–2018, a total of 19,867 adults (aged ≥ 20 years) were examined. Eight anthropometric indexes were calculated. Clinical OA was assessed through participants’ self-reported responses by questionnaires. Multivariable logistic regression analysis and secondary analysis such as restricted cubic splines (RCS), receiver operating characteristic (ROC), decision curve analysis (DCA) and the area under the curve (AUC) analysis were employed to investigate the associations between anthropometric indexes and clinical OA.

**Results:**

The average age of the participants was 46.94 and 49.98% were female. Multivariable logistic regression analysis demonstrated significant associations between all indexes and clinical OA, especially BMI (per 1 standard deviation [SD], odd ration [OR] [95% Confidence interval [CI]] = 1.52[1.40, 1.66]), WTI (OR [95%CI] = 1.50[1.36, 1.65]) and WHtR (OR [95%CI] = 1.50[1.36, 1.64]). Latent profile analysis showed higher indexes could increase clinical OA risk. Additionally, AUC of WWI was the highest, at 0.6724, and DCA indicated that net profit of WWI was higher than other indexes when threshold was below 25%. The results of subgroup analysis proved the robustness of the findings in different sub-populations.

**Conclusion:**

Eight anthropometric indexes related to obesity were all significantly positively associated with clinical OA. Particularly, non-hematological indexes such as WWI and WHtR may show better efficacy in predicting and interventions for clinical OA outcomes, indicating their potential as the preferred strategy for early detection and management of clinical OA.

**Supplementary Information:**

The online version contains supplementary material available at 10.1186/s40001-025-03131-z.

## Introduction

Clinical Osteoarthritis (OA) is a degenerative disease precipitated by a variety of factors, with joint pain as its hallmark symptom and accompanied by fibrosis, osteophyte formation, ulceration, and loss of cartilage, which is the most common form of arthritis, as documented in recent literature [1–3]. Patients with clinical OA frequently experience pain, stiffness, deformity, and a loss of functionality. Moreover, OA is the main cause of disability in the elderly, and the increasing trend of obesity may exacerbate this situation [4]. According to a recent epidemiological study published in 2020, clinical OA affects 3.8% of the global population, which equates to approximately 250 million individuals [5]. The prevalence of clinical OA is projected to increase significantly by 2050 compared to 2020 [6], a trend closely linked to the rise in obesity rates [7]. Therefore, timely identification and intervention in clinical OA cases are crucial to mitigate the disease's prevalence.

Obesity and dyslipidemia are implicated in the pathogenesis of clinical OA [8, 9], and patients with clinical OA have higher rates of dyslipidemia and obesity [10]. Consequently, the rise in obesity rates plays a significant role in the increasing prevalence of clinical OA. Obesity, a medical condition characterized by an excessive accumulation of body fat that leads to metabolic and physiological disruptions [11], is increasingly prevalent in the United States, with nearly half of the population having a body mass index (BMI) of 30 or higher [12]. The association between obesity and clinical OA is well-established, with a higher BMI significantly associated with the development of knee and hip clinical OA [13]. Inflammation and immune responses in adipose tissue due to obesity can lead to local and systemic metabolic issues [14]. While BMI is a commonly used anthropometric measure to define obesity, it does not adequately account for the quantity of visceral fat or dyslipidemia, nor does it consider patterns of body fat distribution [15, 16]. To address these limitations, researchers have proposed novel anthropometric tools that better reflect these characteristics, such as the weight-adjusted-waist index (WWI), waist–height ratio (WHtR), lipid accumulation product (LAP), visceral adiposity index (VAI), triglyceride-glucose index (TyG), body roundness index (BRI), and waist triglyceride index (WTI) [17–20]. The mechanism of these indexes has been well explained in previous studies, but their association with OA has not been compared and verified.

Although numerous studies have explored the correlation between various anthropometric indexes and clinical OA [21–25], few have compared the predictive capabilities of these indexes for clinical OA patients. Given the multitude of proposed anthropometric indexes, this research gap is particularly evident. Moreover, confirming the association between various anthropometric indexes and clinical OA necessitates a large population sample to validate the inferred conclusions. Therefore, this study aims to investigate the relationship between anthropometric indexes (BMI, BRI, WWI, WHtR, TyG, LAP, VAI, and WTI) and clinical OA, and to compare their predictive capabilities for clinical OA patients, with the goal of identifying the most accurate predictive indexes.

## Materials and methods

### Data sources

This study leveraged data from the National Health and Nutrition Examination Survey (NHANES), encompassing seven cycles from 2005 to 2018. NHANES, an ongoing survey, employs a comprehensive multi-stage probability sampling approach to select a representative sample of the U.S. population, with a focus on assessing the health and nutritional status of U.S. adults and children. The NHANES protocol has been approved by the Institutional Review Board of the National Center for Health Statistics (NCHS), and all participants provided written informed consent [26].

## Study design and population

**Fig. 1:**
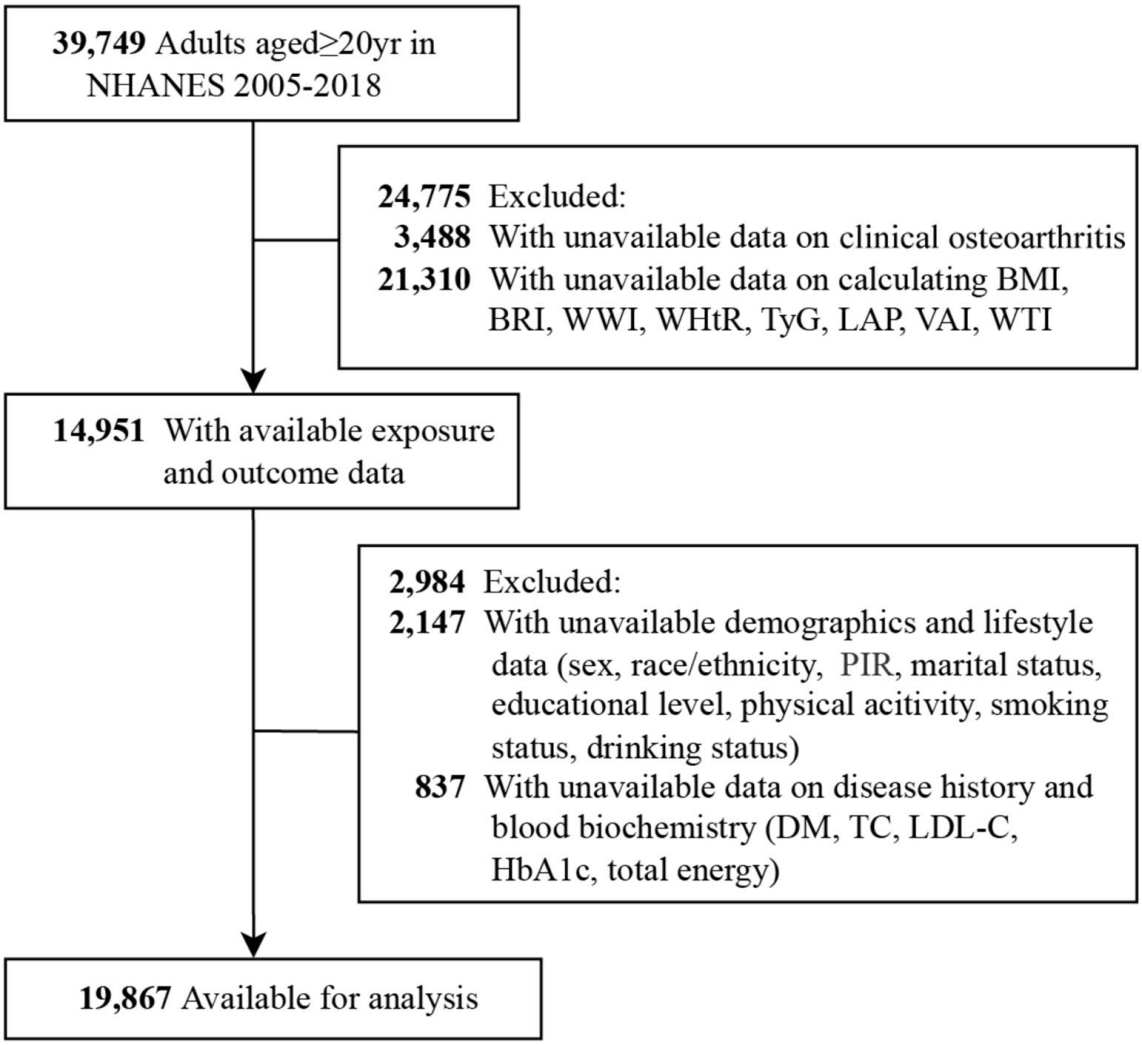
Flowchart of inclusion and exclusion criteria among aged ≥ 20 yr American adults from NHANES 2005–2018

This study utilizes data from the NHANES database, collected from 2005 to 2018, initially comprising 39,749 participants aged over 20 years. Subjects were excluded under the following conditions: missing clinical OA data(*n* = 3,488), inability to calculate BMI, BRI, WWI, WHtR, TyG, LAP, VAI or WTI (missing data on total triglyceride [TG], high-density lipoprotein cholesterol [HDL-C], waist circumference [WC], weight, height, or fasting plasma glucose [FPG]) (*n* = 21,310), and missing covariates (*n* = 2,984). Following these exclusions, the analysis includes 19,867 participants with complete data sets, as shown in Fig. 1.

## Assessment of eight anthropometric indexes

In this study, anthropometric indexes included non-hematological indexes including BMI, BRI, WWI, WHtR, and hematological indexes including TyG, LAP, VAI and WTI. Each participant was interviewed at home and underwent a physical examination at the mobile examination center (MEC). They fasted for at least 9 h before the examination [27]. Height and weight were measured using standardized protocols to calculate BMI, which was rounded to the nearest one-tenth. Waist circumference was measured with a non-elastic tape at the end of normal expiration, with the participant standing naturally, and the value was rounded to the nearest one-tenth of a centimeter [28].

The BMI was calculated using the formula [18]:

$$BMI = \frac{Weight(kg) }{{Height(m) }^{2}}$$

The BRI was calculated using the formula [29]:

$$BRI=364.2 - 365.5\times \sqrt{(1 - \frac{(WC(m) /({2\pi ))}^{2}}{ {\left(0.5 \times Height\left(m\right)\right)}^{2}})}$$

The WWI was calculated using the formula [19]:

$$WWI =\frac{WC(cm)}{\sqrt{Weight(kg)}}$$

The WHtR was calculated using the formula [18]:

$$WHtR =\frac{WC(cm)}{Height(cm)}$$

The TyG was calculated using the formula [30]:

$$\mathit{TyG}=\text{ln}(\frac{TG(mg/dL) \times FPG(mg/dL)}{2})$$

The LAP was calculated using the formula [31]:

$$Males:LAP =\left(WC\left(cm\right)-65\right)\times TG(mmol/L)$$

$$Females:LAP =\left(WC\left(cm\right)-58\right)\times TG(mmol/L)$$

The VAI was calculated using the formula [32]:

$$Males: VAI=\frac{WC(cm)}{39.68+(1.88*BMI(kg/{m}^{2}))}\times \frac{TG(mmol/L)}{1.03}\times \frac{1.31}{HDL-C(mmol/L)}$$

$$Females: VAI=\frac{WC(cm)}{36.58+(1.89*BMI(kg/{m}^{2}))}\times \frac{TG(mmol/L)}{0.81}\times \frac{1.52}{HDL-C(mmol/L)}$$

The WTI was calculated using the formula [33]:

$$WTI = \text{ln}(\frac{TG(mg/dL) \times WC(cm)}{2}$$

## Assessment of clinical osteoarthritis

In epidemiological research, the standard criterion for identifying clinical OA typically involves individuals'self-reported diagnoses, as confirmed by medical professionals, and is commonly gathered by questionnaires [34]. Studies have demonstrated a significant degree of concordance between self-reported clinical OA and that which is clinically diagnosed [35, 36]. The initial inquiry posed to participants is,"Have you ever been informed by a physician or another healthcare professional that you have arthritis?"Those who respond affirmatively are then directed to the subsequent question,"What type of arthritis was it diagnosed as?"Participants who specify"Osteoarthritis or degenerative arthritis"are considered to have been diagnosed with clinical OA [37, 38].

## Covariates

Based on previous and recommendations of relevant studies, this study comprehensively considered a range of covariates, encompassing both demographic and biochemical variables [39–41]. These include age, sex, race/ethnicity, poverty income ratio (PIR), marital status, education level, physical activity (PA) duration, smoking status, drinking status, history of DM, total cholesterol, LDL-cholesterol (LDL-C), glycated hemoglobin (HbA1c) levels, and total energy intake. Sex and race/ethnicity data were self-reported and categorized into five groups: Mexican Americans, non-Hispanic Black Americans, other Hispanic Americans, non-Hispanic White Americans, and other races, including individuals with multiracial backgrounds [42]. Marital status was classified into married, unmarried (including never married; cohabiting with a partner; widowed; divorced; separated; or other) [43]. Education level was stratified into three categories: less than high school, high school or equivalent, and more than high school. Smoking and drinking status were divided into current smoker/drinker and non-smoker/non-drinker [44]. Physical activity was quantified based on the weekly duration of moderate-intensity exercise [45]. The history of diabetes mellitus (DM) was self-reported, and total energy intake was calculated through dietary recall surveys. The specific extraction process for the remaining blood indicators can be found on the NHANES website [46]. In the study, age, PIR, PA time, total energy intake, and serum indicators were treated as continuous variables. Additionally, for subgroup analysis, age was stratified into three groups: 20–39 years, 40–59 years, and 60 years and above.

## Statistical analysis

Considering the complex sampling design and weights, the analysis adhered to the NHANES Analytic Guidelines [46]. Participant characteristics are presented using means (standard errors, SE) for continuous variables and frequencies (percentages) for categorical variables. Continuous data with a normal distribution were analyzed using *t*-tests, while non-normal data were assessed with the Wilcoxon rank-sum test for complex survey samples. Categorical data were evaluated using chi-square tests, with Rao & Scott's second-order correction applied [47, 48].

Multivariable logistic regression analysis was conducted to explore the association between anthropometric indexes and clinical OA. Anthropometric Indexes were entered as both continuous variables (expressed as odds ratios [OR] with 95% confidence intervals [95% CI]) and categorical variables (divided into quartiles). Continuous variables were analyzed per 1 standard deviation (SD) unit after normalization. The crude model was unadjusted, while the adjusted model controlled for all covariates. Trend tests were performed to assess the transition from continuous to categorical variables.

Then, we used the population attributable fraction (PAF) to estimate the proportion of clinical OA which could be avoided if exposure (anthropometric indexes) were eliminated. PAF was calculated using the relative risk from anthropometric indexes (risk of clinical OA for the exposed divided by risk for the non‐exposed) and population prevalence of high anthropometric indexes exposure (proportion exposed) [49]. And the exposures were classified into binary variables based on the cutoff values of Q1 and Q2. Additionally, violin plots depicted anthropometric index variations across groups. To assess the potential non-linear relationship between obesity index and clinical OA, we employed the restricted cubic spline (RCS) method. RCS allows us the flexibility to fit data and capture changes in risk trends. In the analysis, we identify the locations of nodes through a model selection process, which are determined based on the characteristics of the data to ensure that changes in risk trends can be accurately reflected [50].

**Fig. 2:**
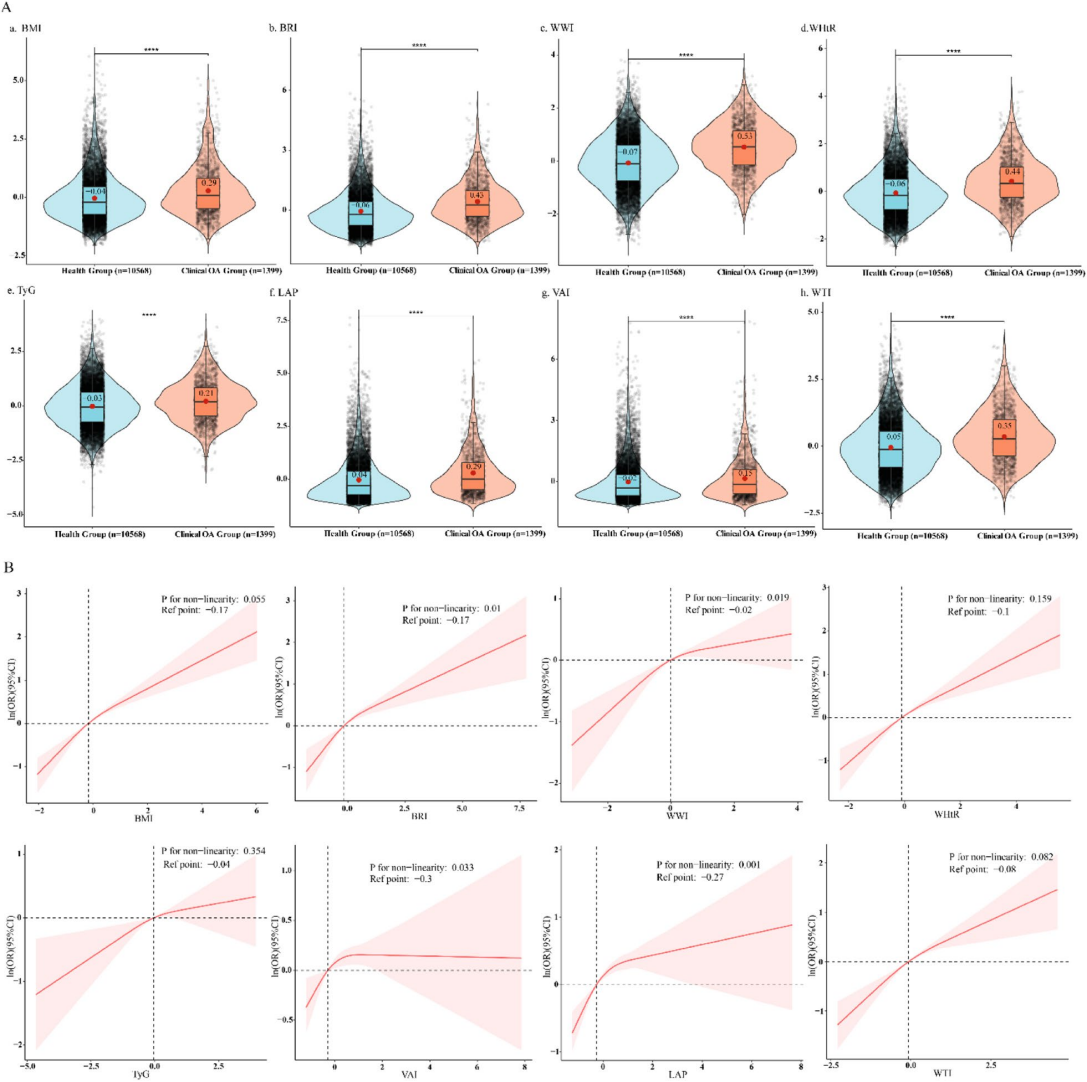
Association of eight anthropometric indexes with prevalence of clinical osteoarthritis estimated by violin plots and restricted cubic splines.^a^ **A** Violin plots^.^ **B** Restricted cubic splines. *BMI* body mass index, *BRI* body roundness index, *CI* confidence interval, *DM* diabetes mellitus, *FPG* fasting plasma glucose, *NHANES* National Health and Nutrition Examination Survey, *HbA1c* hemoglobin A1C, *HDL-C* high-density lipoprotein cholesterol, *LAP* lipid accumulation product, *LDL-C* low-density lipoprotein cholesterol, *OA* osteoarthritis, *PA* physical activity, *PIR* poverty income rate, *RCS* restricted cubic splines, *SD* standard deviation, *SE* standard error, *TC* total cholesterol, *TG* triglyceride, *TyG* triglyceride-glucose index, *VAI* visceral adiposity index, *WC* waist circumference, *WHtR* waist–height ratio, *WTI* waist triglyceride index, *WWI* weight-adjusted-waist index. ^a^. Model was adjusted for age, sex, race/ethnicity, education level, marital status, smoking status, drinking status, PIR, PA time, HbA1c, TC, LDL-C, albumin level, total energy and DM

**Fig. 3:**
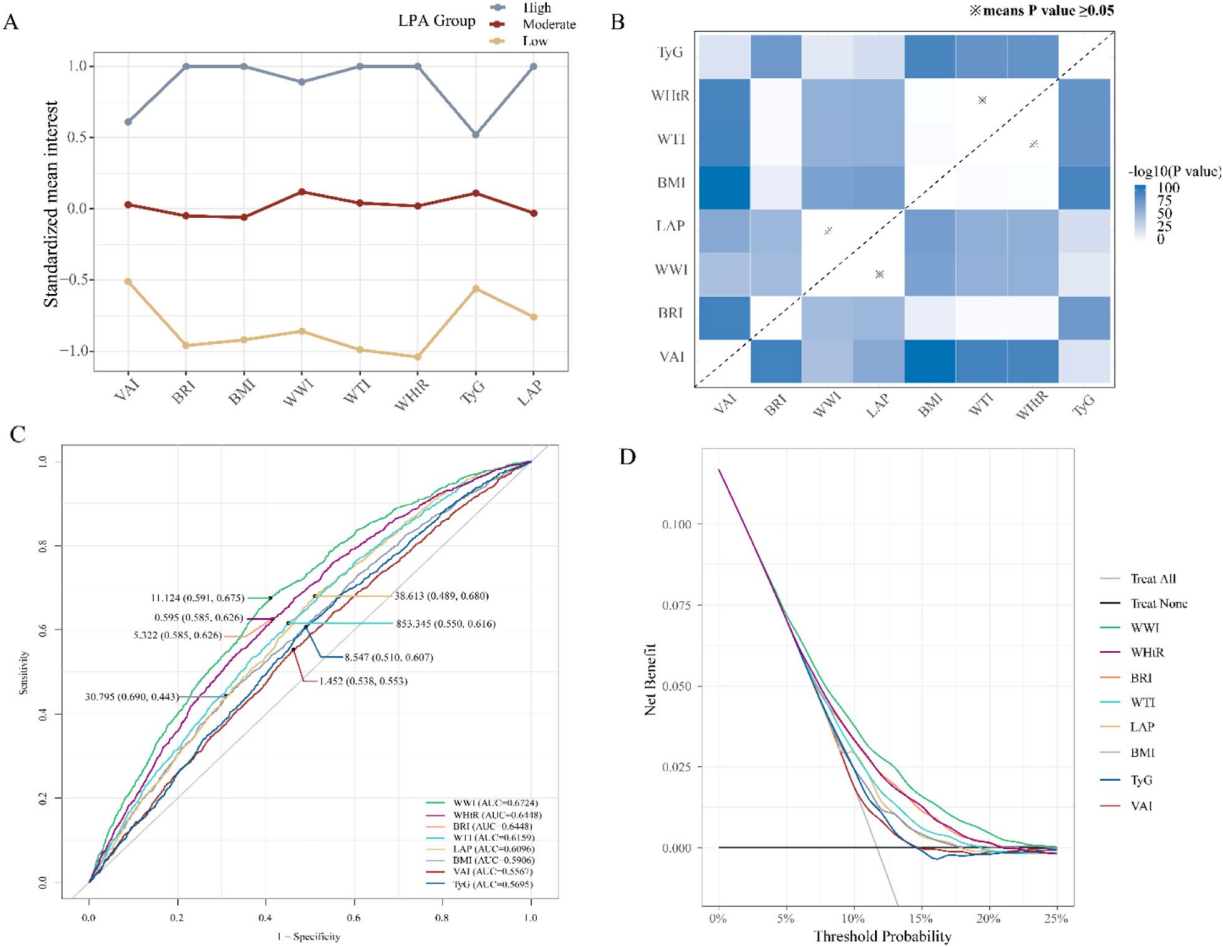
Comparison of eight anthropometric indexes associated with prevalence of clinical osteoarthritis estimated by LPA, bootstrapped estimates, ROC and DCA. **A** Line chart of LPA for identifying potential features of all indexes and grouping them. **B** Comparison of effect values for each indexes using 2-independent-samples t-test based on bootstrapped estimates (*n* = 100). **C** Weighted ROC curves and AUC for comparing all indexes. **D** Weighted DCA curves for comparing all indexes. *AUC* area under curve, *BMI* body mass index, *BRI* body roundness index, *DCA* decision curve analysis, *LAP* lipid accumulation product, *LPA* latent profile analysis, *OA* osteoarthritis, *ROC* receiver operating characteristic, *TyG* triglyceride-glucose index, *VAI* visceral adiposity index, *WHtR* waist–height ratio, *WTI* waist triglyceride index, *WWI* weight-adjusted-waist index

In the secondary analysis, latent profile analysis (LPA) was a Gaussian finite mixture modeling method for identifying different clusters [51]. LPA was used to identify potential characteristics based on all eight continuous anthropometric index components and determine the number of profiles based on the fit of the model. Therefore, the overall index level of participants was divided into three groups: low, moderate, and high, as shown in Fig. 2[52]. To compare the effect values of eight indexes on clinical OA, we used a 2-independent sample t test based on bootstrap estimation (*n* = 100) shown in Fig. 3B [53]. We also used the receiver operating characteristic (ROC) curve to compare the predictive value of eight anthropometric indexes for clinical OA and their predictive power was quantified by area under the curve (AUC) and the threshold of each index was determined [54]. The Akaike Information Criterion (AIC) and Bayesian Information Criterion (BIC) were calculated to compare the model fit for each index [55]. Furthermore, the calibration plot was used to show the mean predicted probability of outcome against the observed proportion of outcomes based on the clinical OA. The degree of agreement between the predicted probability of the model and the actual observation probability is evaluated by the fitting degree between the drawing point and the ideal calibration line and visual is the best way to evaluate calibration [56]. Decision curve analysis (DCA) was used to calculate the net benefit of each risk threshold probability to compare the clinical value of eight anthropometric indexes. This approach helps to understand the practical implications of using these indexes in a clinical setting by quantifying the net benefit under different threshold probabilities. The net benefit reflects the balance between the true positive results (correctly identifying individuals at risk) and the false positive results (incorrectly identifying individuals at risk). A higher net benefit indicates that the index is more effective in identifying individuals who would benefit from interventions while minimizing unnecessary interventions in those not likely to benefit [57].

To detect potential interactions, subgroup analyses were performed by age group, sex, race/ethnicity, marital status, smoking status, drinking status, and DM. An interaction test was conducted by incorporating a cross-product term into the regression model, aiming to investigate the manner in which the influence of one variable on the outcome variable is contingent upon the varying levels of a second variable [41]. We conducted the sensitivity analyses for DM population to re-evaluate the association of anthropometric indexes with clinical OA and the robustness of the results in the DM population. Furthermore, to avoid bias and outlier-driven conclusions caused by acute metabolic abnormalities, we excluded individuals with extremely high or low biochemical values (upper and lower 1% percentiles) and who are known to have diabetes or long-term use of hypoglycemic and lipid-lowering drugs for sensitivity analyses [58].

All statistical analyses were performed using R (version 4.2.3) and the Free Software Foundation's statistical software (version 1.9.2). Specifically, the"survey"package (version 4.2–1) was used for survey sample analysis, and the"mclust"package (version 6.1) was utilized for LPA. A *P*-value of less than 0.05 was considered statistically significant.

## Results

### Characteristics of the participants

Table 1 displays the characteristics of the 11,967 participants with data available for analysis, representing an estimated 75 million adults from the United States aged ≥ 20 years. The weighted mean age was calculated to be 46.94 (SE, 0.28), and the weighted proportion of females among the participants was 49.98%. Among, 1399 were classified as clinical OA group and 10,568 as health group. Participants in the clinical OA group typically tended to be older, female, Non-Hispanic White, married, having a higher household income, currently drinking alcohol, and having history of DM, higher levels of TC and HbA1c in their blood. Notably, the clinical OA group demonstrated lower physical activity and higher total energy intake. Moreover, the violin plots in Fig. 2A showed the differences in eight anthropometric indexes (BMI, BRI, WWI, WHtR, TyG, LAP, VAI, and WTI) between the different groups. Notably, all these indexes were significantly higher in the clinical OA group compared to the healthy group.

**Table 1:** Characteristics of study population

Characteristics	Total^a^ (*n* = 11,967)	Health Group (*n* = 10,568)	Clinical Osteoarthritis Group (*n* = 1399)	*P* value^b^
Weighted Population, *n*	75,827,658	66,246,187	9,581,471	
Age, mean (SE), years	46.94 (0.28)	44.88 (0.28)	61.19 (0.39)	** < 0.001**
Age group, years, *n* (%)				** < 0.001**
20–39	4144 (36.82%)	4067 (41.34%)	77 (5.59%)	
40–50	4033 (38.27%)	3621 (38.49%)	412 (36.75%)	
≥ 60	3790 (24.91%)	2880 (20.17%)	910 (57.67%)	
Sex, *n* (%)				** < 0.001**
Female	5928 (49.98%)	5034 (47.96%)	894 (63.95%)	
Male	6039 (50.02%)	5534 (52.04%)	505 (36.05%)	
Race/ethnicity, *n* (%)^c^				** < 0.001**
Mexican American	1831 (8.08%)	1719 (8.83%)	112 (2.91%)	
Non-Hispanic Black	2380 (9.91%)	2180 (10.53%)	200 (5.68%)	
Non-Hispanic White	5393 (69.90%)	4495 (67.88%)	898 (83.80%)	
Other Hispanic	1124 (5.15%)	1024 (5.51%)	100 (2.69%)	
Other Race^d^	1239 (6.96%)	1150 (7.25%)	89 (4.91%)	
Marital status, *n* (%)^e^				** < 0.001**
No	5711 (44.00%)	5116 (44.99%)	595 (37.13%)	
Yes	6256 (56.00%)	5452 (55.01%)	804 (62.87%)	
Educational level, *n* (%)				0.054
Less than high school	2672 (14.48%)	2414 (14.79%)	258 (12.32%)	
High school or equivalent	2734 (23.06%)	2418 (23.26%)	316 (21.67%)	
Above high school	6561 (62.46%)	5736 (61.95%)	825 (66.01%)	
PIR, Mean (SE)	3.05 (0.04)	3.02 (0.04)	3.20 (0.08)	**0.006**
Smoking status, *n* (%)				0.095
No	9527 (80.21%)	8377 (79.92%)	1150 (82.26%)	
Yes	2440 (19.79%)	2191 (20.08%)	249 (17.74%)	
Drinking status, *n* (%)				** < 0.001**
No	3632 (24.60%)	3114 (23.84%)	518 (29.81%)	
Yes	8335 (75.40%)	7454 (76.16%)	881 (70.19%)	
PA time (min), Mean (SE)	882.98 (21.85)	917.86 (23.99)	641.81 (35.03)	** < 0.001**
TC (mmol/L), Mean (SE)	4.97 (0.01)	4.95 (0.01)	5.09 (0.03)	** < 0.001**
HbA1c, Mean (SE)	5.59 (0.01)	5.56 (0.01)	5.81 (0.04)	** < 0.001**
LDL-C (mmol/L), Mean (SE)	2.95 (0.01)	2.95 (0.01)	2.95 (0.03)	0.58
DM, *n* (%)				** < 0.001**
No	9614 (85.04%)	8636 (86.44%)	978 (75.35%)	
Yes	2353 (14.96%)	1932 (13.56%)	421 (24.65%)	
Total energy (kcal), Mean (SE)	2190.80 (11.47)	2216.54 (12.81)	2012.86 (30.45)	** < 0.001**
BMI, Mean (SE)	28.86 (0.10)	28.58 (0.11)	30.75 (0.27)	** < 0.001**
BMI, per SD, Mean (SE)	−0.02 (0.01)	−0.06 (0.02)	0.26 (0.04)	** < 0.001**
BRI, Mean (SE)	5.29 (0.04)	5.15 (0.04)	6.27 (0.09)	** < 0.001**
BRI, per SD, Mean (SE)	−0.05 (0.02)	−0.11 (0.02)	0.37 (0.04)	** < 0.001**
WWI, Mean (SE)	10.92 (0.02)	10.86 (0.02)	11.36 (0.03)	** < 0.001**
WWI, per SD, Mean (SE)	−0.10 (0.02)	−0.17 (0.02)	0.43 (0.04)	** < 0.001**
WHtR, Mean (SE)	0.59 (0.00)	0.58 (0.00)	0.63 (0.00)	** < 0.001**
WHtR, per SD, Mean (SE)	−0.06 (0.02)	−0.12 (0.02)	0.38 (0.04)	** < 0.001**
TyG, Mean (SE)	8.56 (0.01)	8.54 (0.01)	8.71 (0.02)	** < 0.001**
TyG, per SD, Mean (SE)	−0.04 (0.02)	−0.07 (0.02)	0.21 (0.04)	** < 0.001**
LAP, Mean (SE)	52.68 (0.73)	50.77 (0.68)	65.90 (2.16)	** < 0.001**
LAP, per SD, Mean (SE)	0.01 (0.02)	−0.04 (0.02)	0.32 (0.05)	** < 0.001**
VAI, Mean (SE)	1.79 (0.02)	1.75 (0.02)	2.04 (0.07)	** < 0.001**
VAI, per SD, Mean (SE)	−0.01 (0.02)	−0.03 (0.01)	0.17 (0.05)	** < 0.001**
WTI, Mean (SE)	850.67 (2.92)	841.74 (3.06)	912.43 (7.10)	** < 0.001**
WTI, per SD, Mean (SE)	−0.02 (0.02)	−0.07 (0.02)	0.34 (0.04)	** < 0.001**

All means and SEs for continuous variables and numbers and percentages for categorical variables were weighted

*BMI* body mass index, *BRI* body roundness index, *CI* confidence interval, *DM* diabetes mellitus, *FPG* fasting plasma glucose, *NHANES* National Health and Nutrition Examination Survey, *HbA1c* hemoglobin A1C, *HDL-C* high-density lipoprotein cholesterol, *LAP* lipid accumulation product, *LDL-C* low-density lipoprotein cholesterol, *OA* osteoarthritis, *OR* odd ratio, *PA* physical activity, *PIR* poverty income rate, *SD* standard deviation, *SE* standard error, *TC* total cholesterol, *TG* triglyceride, *TyG* triglyceride-glucose index, *VAI* visceral adiposity index, *WC* waist circumference, *WHtR* waist–height ratio, *WTI* waist triglyceride index, *WWI* weight-adjusted-waist index

^a^ The sample size (*n*) is unweighted but the % is accounted for complex sampling design

^b^ p-values were obtained using chi-squared test with Rao & Scott's second-order correction for categorical variables, and Wilcoxon rank-sum test for continuous variables, all were accounted for complex sampling design

^c^ Race and ethnicity were self-reported

^d^ Includes multi-racial participants. NHANES does not provide a detailed list of all races and ethnicities

^e^ Married status was classified into: No (Never married; Living with partner; Widowed; Divorced; Separated individuals); Yes (Married)

## Association of eight anthropometric indexes with clinical osteoarthritis

After adjusting for confounding factors, every 1 SD increase in the eight anthropometric indexes (BMI, BRI, WWI, WHtR, TyG, LAP, VAI, and WTI) was associated with an increased risk of clinical OA ranging from 10 to 52% (OR[95%CI]: for per 1 SD increase: BMI 1.52[1.40, 1.66]; BRI 1.45[1.33, 1.59]; WWI 1.25[1.13, 1.39]; WHtR 1.50[1.36, 1.64]; TyG 1.18[1.06, 1.31]; LAP 1.27[1.18, 1.38]; VAI 1.10[1.02, 1.18], and WTI 1.50[1.36, 1.65]). In the quartile analysis, the highest quartile (Q4) for all indexes demonstrated a significant increase in clinical OA risk compared to the first quartile (Q1), with a significant trend observed in the trend test. Additionally, LPA showed that participants with high overall index level (high group) had a 169% increased risk of clinical OA compared to the low group (OR [95% CI]: 2.69[2.07, 3.51]) (Fig. 3A, Table 2).

**Table 2:** Association and Population attributable fraction (PAF) of eight anthropometric indexes with the prevalence of clinical osteoarthritis

	Crude Model^a^		PAF^b^	Adjusted Model^c^		PAF
	OR (95%CI)	*P* value	Q1 vs Q2-Q4	OR (95%CI)	*P* value	Q1 vs Q2-Q4
BMI, per SD	1.33(1.24, 1.44)	** < 0.001**	30.34%	1.52(1.40, 1.66)	** < 0.001**	30.60%
BMI Group						
Q1	Reference			Reference		
Q2	1.66(1.32, 2.08)	** < 0.001**		1.62(1.24, 2.11)	** < 0.001**	
Q3	1.61(1.28, 2.02)	** < 0.001**		1.75(1.33, 2.29)	** < 0.001**	
Q4	2.39(1.89, 3.02)	** < 0.001**		3.03(2.33, 3.93)	** < 0.001**	
*P* for trend		** < 0.001**			** < 0.001**	
BRI, per SD	1.51(1.41, 1.62)	** < 0.001**	50.30%	1.45(1.33, 1.59)	** < 0.001**	33.54%
BRI Group						
Q1	Reference			Reference		
Q2	2.07(1.64, 2.61)	** < 0.001**		1.56(1.20, 2.04)	**0.001**	
Q3	2.68(2.13, 3.39)	** < 0.001**		1.91(1.44, 2.54)	** < 0.001**	
Q4	4.30(3.42, 5.41)	** < 0.001**		3.03(2.29, 4.00)	** < 0.001**	
*P* for trend		** < 0.001**			** < 0.001**	
WWI, per SD	1.88(1.75, 2.03)	** < 0.001**	54.50%	1.25(1.13, 1.39)	** < 0.001**	17.05%
WWI Group						
Q1	Reference			Reference		
Q2	2.13(1.67, 2.71)	** < 0.001**		1.36(1.06, 1.74)	**0.02**	
Q3	3.19(2.55, 3.99)	** < 0.001**		1.48(1.14, 1.92)	**0.004**	
Q4	5.27(4.20, 6.60)	** < 0.001**		1.68(1.25, 2.25)	** < 0.001**	
*P* for trend		** < 0.001**			**0.002**	
WHtR, per SD	1.58(1.46, 1.70)	** < 0.001**	50.29%	1.50(1.36, 1.64)	** < 0.001**	33.54%
WHtR Group						
Q1	Reference			Reference		
Q2	2.07(1.64, 2.61)	** < 0.001**		1.56(1.20, 2.04)	**0.001**	
Q3	2.68(2.13, 3.39)	** < 0.001**		1.91(1.44, 2.54)	** < 0.001**	
Q4	4.30(3.42, 5.41)	** < 0.001**		3.03(2.29, 4.00)	** < 0.001**	
*P* for trend		** < 0.001**			** < 0.001**	
TyG, per SD	1.32(1.23, 1.42)	** < 0.001**	25.68%	1.18(1.06, 1.31)	**0.003**	6.21%
TyG Group						
Q1	Reference			Reference		
Q2	1.27(1.00, 1.61)	0.05		1.00(0.77, 1.31)	0.97	
Q3	1.89(1.49, 2.40)	** < 0.001**		1.47(1.12, 1.91)	**0.01**	
Q4	2.05(1.64, 2.57)	** < 0.001**		1.38(1.01, 1.88)	**0.04**	
*P* for trend		** < 0.001**			**0.005**	
LAP, per SD	1.33(1.25, 1.41)	** < 0.001**	42.60%	1.27(1.18, 1.38)	** < 0.001**	25.94%
LAP Group						
Q1	Reference			Reference		
Q2	1.72(1.33, 2.22)	** < 0.001**		1.32(0.99, 1.74)	0.06	
Q3	2.49(1.94, 3.21)	** < 0.001**		1.89(1.40, 2.55)	** < 0.001**	
Q4	3.03(2.39, 3.84)	** < 0.001**		2.22(1.66, 2.96)	** < 0.001**	
*P* for trend		** < 0.001**			** < 0.001**	
VAI, per SD	1.19(1.12, 1.27)	** < 0.001**	18.15%	1.10(1.02, 1.18)	**0.02**	9.53%
VAI Group						
Q1	Reference			Reference		
Q2	1.24(0.97, 1.59)	0.09		1.22(0.93, 1.60)	0.16	
Q3	1.47(1.20, 1.80)	** < 0.001**		1.29(1.01, 1.65)	**0.04**	
Q4	1.83(1.47, 2.27)	** < 0.001**		1.52(1.17, 1.97)	**0.002**	
*P* for trend		** < 0.001**			**0.003**	
WTI, per SD	1.45(1.35, 1.56)	** < 0.001**	41.52%	1.50(1.36, 1.65)	** < 0.001**	30.41%
WTI Group						
Q1	Reference			Reference		
Q2	1.84(1.47, 2.30)	** < 0.001**		1.53(1.18, 1.98)	**0.001**	
Q3	2.16(1.71, 2.73)	** < 0.001**		1.85(1.37, 2.52)	** < 0.001**	
Q4	3.17(2.53, 3.97)	** < 0.001**		2.87(2.20, 3.74)	** < 0.001**	
*P* for trend		** < 0.001**			** < 0.001**	
LPA Group^d^						
Low	Reference			Reference		
Moderate	2.24(1.84, 2.71)	** < 0.001**		1.86(1.45, 2.37)	** < 0.001**	
High	3.26(2.60, 4.10)	** < 0.001**		2.69(2.07, 3.51)	** < 0.001**	
*P* for trend		** < 0.001**			** < 0.001**	

*BMI* body mass index, *BRI* body roundness index, *CI* confidence interval, *DM* diabetes mellitus, *FPG* fasting plasma glucose, *NHANES* National Health and Nutrition Examination Survey, *HbA1c* hemoglobin A1C, *HDL-C* high-density lipoprotein cholesterol, *LAP* lipid accumulation product, *LDL-C* low-density lipoprotein cholesterol, *LPA* latent profile analysis, *OA* osteoarthritis, *OR* odd ratio, *PA* physical activity, *PAF* Population attributable fraction, *PIR* poverty income rate, *SD* standard deviation, *SE* standard error, *TC* total cholesterol, *TG* triglyceride, *TyG* triglyceride-glucose index, *VAI* visceral adiposity index, *WC* waist circumference, *WHtR* waist–height ratio, *WTI* waist triglyceride index, *WWI* weight-adjusted-waist index

^a^ Crude model: No adjustment for confounding factors

^b^ Using Population Attribution Fraction (PAF) to estimate the proportion of patients with clinical OA who can be reduced by eliminating exposures. And the exposures were classified into binary variables based on the cutoff values of Q1 and Q2

^c^ Adjusted for age, sex, race/ethnicity, education level, marital status, smoking status, drinking status, PIR, PA time, HbA1c, TC, LDL-C, albumin level, total energy and DM

^d^ Identify potential features of eight continuous anthropometric indexes through latent profile analysis (LPA) using *R* packages “mclust”, and group them according to their potential features: Low, Moderate, High Group

In the RCS analysis, a significant linear positive association with clinical OA was observed only for the indexes BMI, WHtR, TyG, and WTI (*P* for non-linearity > 0.05) (Fig. 2B).In contrast, for BRI, WWI, VAI, and LAP, different patterns were identified. For these four indexes, a significant non-linear relationship was observed (P for non-linearity < 0.05). The reference points for BRI, WWI, VAI, and LAP are located at  -0.17,  -0.02,  -0.3, and  -0.27, respectively. This indicates that the change in the prevalence of clinical OA is not constant as these values increase. As depicted in the figure, after their respective reference points, the curves for these indexes show a trend of either a slower increase or even a decrease. This non-constant change may imply complex interaction mechanisms or threshold effects between these variables and clinical OA pathogenesis.

In addition, PAF results suggested that eliminating the effect of high indexes levels may reduce the proportion of clinical OA patients from 6.21% to 33.54%. Notably, the lowest reduction in the proportion of clinical OA patients was observed for WHtR and BRI, which were both 33.54% (Table 2).

In the secondary analysis, based on the bootstrap method, the comparison of effect values showed that except LAP and WWI, WHtR and WTI, there were significant differences between the effect values of other indicators (Fig. 3B). It demonstrated that BMI had the highest effect value, followed by WHtR, WTI and BRI, while VAI has the lowest effect value. Figure 3C displays the ROC curve demonstrated that the WWI has a robust predictive capacity in the Fully adjusted weighted model, with an AUC of 0.6724 and the threshold of BMI, BRI, WWI, WHtR, TyG, LAP, VAI, and WTI were 30.795, 5.322, 11.124, 0.595, 8.547, 38.613, 1.452 and 853.345. Among them, the AIC/BIC of WWI and WHtR was the smallest, which were 8586.9/8611.8 and 8780.8/8804.9 respectively, indicating that their model fitting quality was the best (Supplementary Table 2). Supplementary Fig. 2 describes the Calibration plot showing the mean predicted probability of outcome against the observed proportion of outcomes based on the clinical OA. It was observed that the plotting points of each indicator were in close agreement with the ideal curve, indicating that the predicted probabilities were basically consistent with the actual observed probabilities, and the performance and applicability of the model could be evaluated more completely. And the DCA indicates that the net benefit of interventions based on WWI for reducing the risk of clinical OA was significantly higher than that of other indexes when the threshold probability was below 25% (Fig. 3D). This suggests that, in clinical settings, using WWI to guide interventions could be more beneficial than using other indexes when the probability of OA is estimated to be below this threshold.

## Subgroup analyses and sensitivity analyses

Supplementary Table 1 presents the results of a sensitivity analysis conducted within the DM population. In this analysis, no significant associations were observed among the four blood-related indexes (TyG, LAP, VAI, and WTI). In contrast, the non-blood-related indicators (BMI, WHtR, WWI, BRI) showed robust associations with OA. This suggests that in the DM population, additional influential factors may need to be considered for a more comprehensive understanding of the underlying associations. The blood-related indexes might be affected by the unique metabolic profile of DM patients, which could mask their association with OA. Our analysis highlights the importance of considering the distinct characteristics of different populations when interpreting the relationship between anthropometric indexes and OA risk. It also indicates that non-blood-related indicators may serve as more reliable predictors of clinical OA across diverse populations, including those with DM. Even after excluding the 1% outliers, the results were consistent with the population-wide results, suggesting that the robustness of the results is not driven by outliers (Supplementary Table 3). After excluding those who are known to have diabetes or long-term use of hypoglycemic and lipid-lowering drugs, the results remained robust overall (Supplementary Table 4).

Supplementary Fig. 1 depicts the associations between various anthropometric indexes and clinical OA across different baseline subgroups. In the multivariable analysis, stratification was performed according to sex, race/ethnicity, education level, marital status, smoking status, drinking status, and DM. Notably, significant interaction effects were observed between age and WWI associated with clinical OA, and DM and TyG or LAP associated with clinical OA (*p* < 0.05). These findings suggested that the associations between anthropometric indexes and clinical OA may vary depending on these specific factors, highlighting the importance of considering these variables in the analysis.

## Discussion

In this nationally representative cross-sectional study, after adjusting for confounding factors such as demographics, lifestyle, and serum indicators, it was found that participants with higher anthropometric indexes had an increased risk of clinical OA. Interventions targeting the WHtR could lead to a significant reduction in the proportion of clinical OA-affected population. Furthermore, the analysis of the ROC and DCA curves revealed that the WWI demonstrated promising clinical utility in predicting clinical OA, with favorable performance metrics. This suggests that WWI and WHtR may perform better in predicting and intervening in clinical OA outcomes, suggesting that these non-blood tests are more conducive to becoming an early detection and management of OA.

Emerging evidence suggests that obesity may heighten the risk of developing clinical OA [59]. Previous epidemiological studies have indicated that various anthropometric indexes used to predict obesity play a significant role in clinical OA, showing substantial effects across different types of the condition. For instance, Florent Eymard et al.[60]. Noted that type 2 diabetes mellitus (T2DM) predicts a reduction in joint space width in males with knee OA (KOA). Rehling and colleagues [61] found a close association between clinical OA prevalence and DM. And Jiang et al.[62]. Observed that an increase in BMI significantly raises the risk of KOA. Similar to the results of our subgroup analysis, DM and age may have a potential influence between anthropometric measures and clinical OA. According to Badley et al., combinations of joint sites including the knee are closely linked with BMI [22]. Furthermore, central obesity, indicated by the WHtR, has been significantly correlated with radiographic knee OA (RKOA) events, with a stronger association than BMI [25]. Our study demonstrates that similar conclusions can be made in clinical OA. A clinical practice guideline suggested WHtR has greater discriminatory power over BMI for diabetes and cardiovascular disease [63]. Moreover, WWI's potential in predicting clinical OA has been confirmed by Li et al. [64]. Our study also suggests that WHtR and WWI may have better discriminatory power to clinical OA than other indicators, which can play a role of low economic cost and high benefit in primary care or public health settings. For the cutoff values of the ROC, closer attention may be needed at these optimal values, but this needs to be verified in a wider queue. The cut-off value of blood indicators is basically lower than that of non-blood indicators, especially WWI has the highest sensitivity and specificity among these indicators. The role of this needs to be further explored. Elevated BMI has also been significantly correlated with the onset of knee and hip clinical OA [25]. A large Chinese cohort study also demonstrated a significant association between anthropometric measures such as BMI, WHtR, and BRI with KOA [65], which was an important evidence for the extrapolability of our study. This was consistent with the conclusion in this study that non-blood-related indexes had a more favorable effect. Furthermore, our previous studies have shown that there is a significant positive association between Tyg and OA in different populations [66]. On the basis of their findings, this study further investigated the association between different indicators and clinical OA and suggests that screening for clinical OA can be achieved through non-blood tests and is more effective than blood tests, which is of great clinical significance. By incorporating these simple non-invasive measures into routine medical examinations, interventions such as lifestyle changes, exercise prescriptions and dietary counselling can be implemented in a timely manner. In addition, in the public health arena, the use of WWI and WHtR for population-wide clinical OA screening has the potential to help develop targeted prevention strategies and health promotion initiatives to reduce the burden of care in the community. And community-based screening for clinical OA in people with different ages and chronic diseases may be more effective.

For the four indicators related to blood indicators, LAP serves as a valuable biomarker for confirming obesity exposure, which increases with waist circumference and triglyceride levels [24, 67, 68]. WWI is considered more accurate than waist circumference and BMI for assessing the health risks associated with obesity [69, 70]. Huang et al.[21]. Suggested that the TyG may be a valuable predictor for clinical OA. Yan et al. [71]. Elucidated that in Americans under 60 with average weight and without diabetes, the TyG index correlates positively with arthritis. Moreover, TyG combined with WHtR, can further stratify and predict diabetes risk [72]. However, in this study, they did not reflect a better effect than non-blood-related measures, indicating that further prospective studies are needed to confirm this. And our subgroup analysis results, consistent with previous studies, show significant interactions in subgroups stratified by sex, age, drinking status, and DM, indicating that this positive correlation may apply to many demographic backgrounds. In our study analysis, the significant positive correlation between various anthropometric indexes and clinical OA aligns with previous findings, further demonstrating the robustness of the results.

The super ior performance of WWI and WHtR in predicting OA can be attributed to several factors. Firstly, these indices offer a more precise assessment of visceral fat distribution. WWI, by integrating weight and waist circumference, provides a sensitive measure of visceral fat, which is not only a source of mechanical load on joints but also a source of pro-inflammatory cytokines that directly contribute to OA pathogenesis [23, 73, 74]. WHtR, by standardizing abdominal fat through the waist-to-height ratio, avoids the limitations of BMI and directly reflects the local impact of central obesity on joints [75–77]. Secondly, while visceral fat distribution is linked to metabolic dysfunction, the advantage of WWI and WHtR may stem more from their ability to quantify mechanical load [74]. In contrast, metabolic indices like TyG and LAP, although capable of reflecting insulin resistance and lipid accumulation, may have their associations with OA diluted by systemic metabolic factors, as OA's local inflammatory mechanisms may rely more on the physical and paracrine effects of fat distribution [66, 77]. Furthermore, the weaker performance of hematological indices such as TyG and LAP compared to non-hematological ones can be explained by several factors. Blood-based measurements are subject to variability due to diet, testing time, and individual metabolic status, whereas morphological indicators like waist circumference are more stable [78]. OA development may be more dependent on the local inflammatory microenvironment of adipose tissue than on systemic metabolic disorders. Leptin and adiponectin released from visceral fat can directly act on articular cartilage, which cannot be captured by TyG and other indicators [79]. In summary, the superiority of WWI and WHtR likely stems from their comprehensive assessment of visceral fat distribution and mechanical load, while the limitations of hematological indices reflect the local nature of OA's pathological mechanisms and the inherent variability of blood-based measurements. Future research combining multi-omics data is needed to further explore the interplay between fat distribution, metabolic dysfunction, and OA.

The pathophysiological mechanisms by which obesity induces clinical OA are multifaceted. Clinical OA involves synovium, tendons, muscles, ligaments, subchondral bone, adipose tissue, and articular cartilage, with inflammation playing a crucial role in the interaction of joint tissues [80]. Understanding how obesity contributes pathophysiology to OA progression to guide the development of screening indicators is important for health policy makers [81].

For the roles of adipokines in clinical OA, adipose tissue-derived factors, or adipokines, have been implicated in the pathogenesis of clinical OA. Specifically, adipokines such as leptin, adiponectin, and resistin have been shown to influence the development and progression of clinical OA [82, 83]. Leptin, for instance, has been found to stimulate the expression of matrix-degrading enzymes and pro-inflammatory mediators in chondrocytes through the NF-κB signaling pathway. Adiponectin, on the other hand, has anti-inflammatory and anti-apoptotic properties and is closely related to the development and progression of clinical OA [84]. It has been shown to increase the expression of matrix metalloproteinases (MMPs) and inducible nitric oxide synthase (iNOS) in human OA chondrocytes, leading to cartilage degradation [85]. Regarding the systemic inflammation, and metabolic dysregulation, a study by Martine Duclos et al.[86]. Found that elevated blood sugar levels can lead to inflammation and cartilage degeneration through oxidative stress, the accumulation of inflammatory mediators, and advanced glycation end products. Moreover, the degeneration of articular cartilage in OA patients may be related to the dysfunction of mitochondrial homeostasis [87]. Activation of the AMPK pathway enhanced mitochondrial proliferation and biogenesis, and significant cartilage regeneration was promoted by mitochondrial treatment in the OA mouse model [87, 88]. Recent evidence suggests that the relationship between obesity and RKOA may also be driven by metabolic factors, such as the release of pro-inflammatory adipokines like leptin [89]. Rosa et al.[90]. Reported that chondrocytes express functional insulin receptors in adults. In clinical OA patients, chondrocytes exhibit reduced capacity to enhance glucose transport in response to normal physiological levels of insulin. This impairment in glucose transport may damage energy production and the plasticity functions of chondrocytes, including the synthesis of chondroitin sulfate. And insulin resistance impairs the body's ability to use insulin effectively, leading to reduced bone formation by inhibiting collagen synthesis and osteoblast activity [91, 92]. Ultimately, this could lead to chondrocyte damage and the progression of clinical OA. Li et al. [93] indicated that the NF-κB and NLRP3 signaling pathways synergistically support P2X7-induced chondrocyte extracellular matrix degradation and pyroptosis, suggesting P2X7 as a potential target for clinical OA treatment. Jin et al.[94]. Demonstrated that quercetin can inhibit NF-κB activation in chondrocytes. This inhibition occurs through the activation of the Nrf2/HO-1 signaling pathway, significantly reducing IL-1β-induced inflammatory responses and catabolic processes. Nrf2 also played an important role in osteoclast differentiation and bone resorption [95]. Additionally, VAI is used as a marker of visceral adipose tissue dysfunction [32]. Studies have shown that visceral fat is an important source of pro-inflammatory cytokines, leading to low-grade chronic metabolic inflammation, potentially causing joint structural damage [96]. These findings suggest that obesity may be an important factor in the progression of clinical OA and may serve as a marker for early screening of clinical OA, which provides a theoretical basis for further large-scale epidemiological studies in the future.

By studying multiple anthropometric measures and comparing blood and non-blood measures, our study has important guiding implications for screening for clinical OA in hospitals and communities. Here are some of the strengths of our study. Firstly, it was the first cross-sectional survey to compare the association between eight anthropometric indexes and clinical OA among U.S. adults, which was of significant importance for clinicians and individuals in daily Life to select the most effective and convenient index for predicting clinical OA. Secondly, the study strictly adhered to the NHANES standards in study design, data collection, and processing, thereby minimizing the potential for non-sampling and measurement errors. Thirdly, the integration of complex sampling weights and careful consideration of sample design ensured a nationally representative sample estimate, representing over 75 million U.S. adults. Additionally, our study conducted LPA for all indexes, which allows for more precise classification based on their potential characteristics and more effectively validates the association between high or low anthropometric indexes and clinical OA. The predictive capabilities of the indexes were further examined using PAF, ROC and DCA curves, providing mutual validation and demonstrating the clinical practicality of intervening in clinical OA treatment.

However, this study was not without its limitations. Firstly, reliance on self-reported clinical OA diagnoses may introduce discrepancies compared to objective reality. But, as previously mentioned, clinical OA information was most collected through questionnaires confirmed by professionals. Patient-reported outcomes were likely to be a future trend due to their cost-effectiveness, convenience, and emphasis on the patient's own experience. Nevertheless, any effort to validate these self-reports against objective criteria, such as radiographic evidence, would be beneficial. Future studies adopting more robust data collection methods, such as international classification of diseases (ICD) diagnostic coding and combining biomarkers (such as synovial fluid analysis) or advanced imaging techniques (high-resolution magnetic resonance imaging, molecular imaging), could enhance the credibility of the research[97, 98]. And adopting hybrid data collection methods (e.g. electronic health record combined with patient self-administered questionnaire) will improve data integrity [99]. Secondly, like other observational studies, our survey lacks the ability to ultimately eliminate all residual or unidentified confounding factors, as well as potential confounding effects caused by measurement errors and assessment of variables. And the cross-sectional design of our study precludes the establishment of temporal relationships and causal inferences between the anthropometric indexes and clinical OA. Because this study used a cross-sectional study, it was not possible to clarify whether exposure (indexes of obesity) occurred before clinical OA, which could easily lead to temporal misalignment and reverse causality bias. To enhance the validity of our findings and address these limitations, future prospective randomized controlled trials or longitudinal studies would be valuable. Such studies could help clarify the temporal sequence and causal relationships, thereby providing a more comprehensive understanding of the associations between anthropometric indexes and clinical OA. Furthermore, there are limitations in predicting clinical OA by hematologic parameters such as TyG and LAP, especially in diabetic patients. In future prospective and large-scale epidemiological studies, the patients with diabetes were stratified by severity, duration, and glycemic control, combined with biomarkers such as HbA1c and improve the testing techniques to achieve a breakthrough. Lastly, due to the inherent nature of observational studies, a definitive causal relationship cannot be established. As the scope of this data study is limited to U.S. adults, further regional studies are needed to determine its applicability to populations in other parts of the world.

## Conclusions

To synthesize the findings, this research highlights a robust positive correlation between a spectrum of anthropometric indexes—BMI, BRI, WWI, WHtR, TyG, LAP, VAI, and WTI—and the risk of clinical OA. Particularly, the WHtR and WWI indexes merit attention for their enhanced capacity to identify individuals at risk for clinical OA. A key benefit of these indexes is their non-invasive approach, which foregoes the need for intrusive diagnostic methods. This attribute not only makes them potent tools for risk stratification but also more clinically viable and patient-centric alternatives. The ease of use and reliability of WHtR and WWI make them top contenders as sentinel indexes for clinical OA management. Moreover, their non-invasive nature is particularly advantageous for integrating into preventive and therapeutic strategies against obesity-related clinical OA, thereby extending their utility across public health campaigns.

## Supplementary Information

Supplementary Material1

## Data Availability

The NHANES data are publicly available at https://wwwn.cdc.gov/nchs/nhanes which is publicly available. The data underlying this article will be shared on reasonable request to the corresponding author.
