# Supplementary material for: Association of eight anthropometric indexes related to obesity with the prevalence of clinical osteoarthritis among American adults: a national cross-sectional study
Source: Eur J Med Res. 2025 Sep 26;30:871. doi: 10.1186/s40001-025-03131-z (PMC12465158; doi:10.1186/s40001-025-03131-z)
Supplement: Supplementary file 1 — Supplementary Material1 [file 40001_2025_3131_MOESM1_ESM.docx]

**Supplementary Materials Files**

To: **Association of Eight Anthropometric Indexes related to Obesity with the Prevalence of Clinical Osteoarthritis among American adults: A National Cross-Sectional Study**

**Supplementary** **Figure 1.** **Subgroups analyses and Forest plots of Association of Eight anthropometric indexes with prevalence of Clinical Osteoarthritis in Different Subgroups.**

**Supplementary Figure 2. Calibration plot showing the mean predicted probability of outcome against the observed proportion of outcomes based on the clinical OA.**

**Supplementary Table 1. Association of Eight anthropometric indexes with the Prevalence of Clinical Osteoarthritis in DM groups.**

**Supplementary Table 2. The AUC and thresholds of weighted ROC curves for comparing all indexes.**

**Supplementary Table 3. Association of Eight anthropometric indexes with the Prevalence of Clinical Osteoarthritis after excluding the upper and lower 1% extremes.**

**Supplementary Figure 1. Subgroups analyses and Forest plots of Association of Eight anthropometric indexes with prevalence of Clinical Osteoarthritis in Different Subgroups^a^**

**
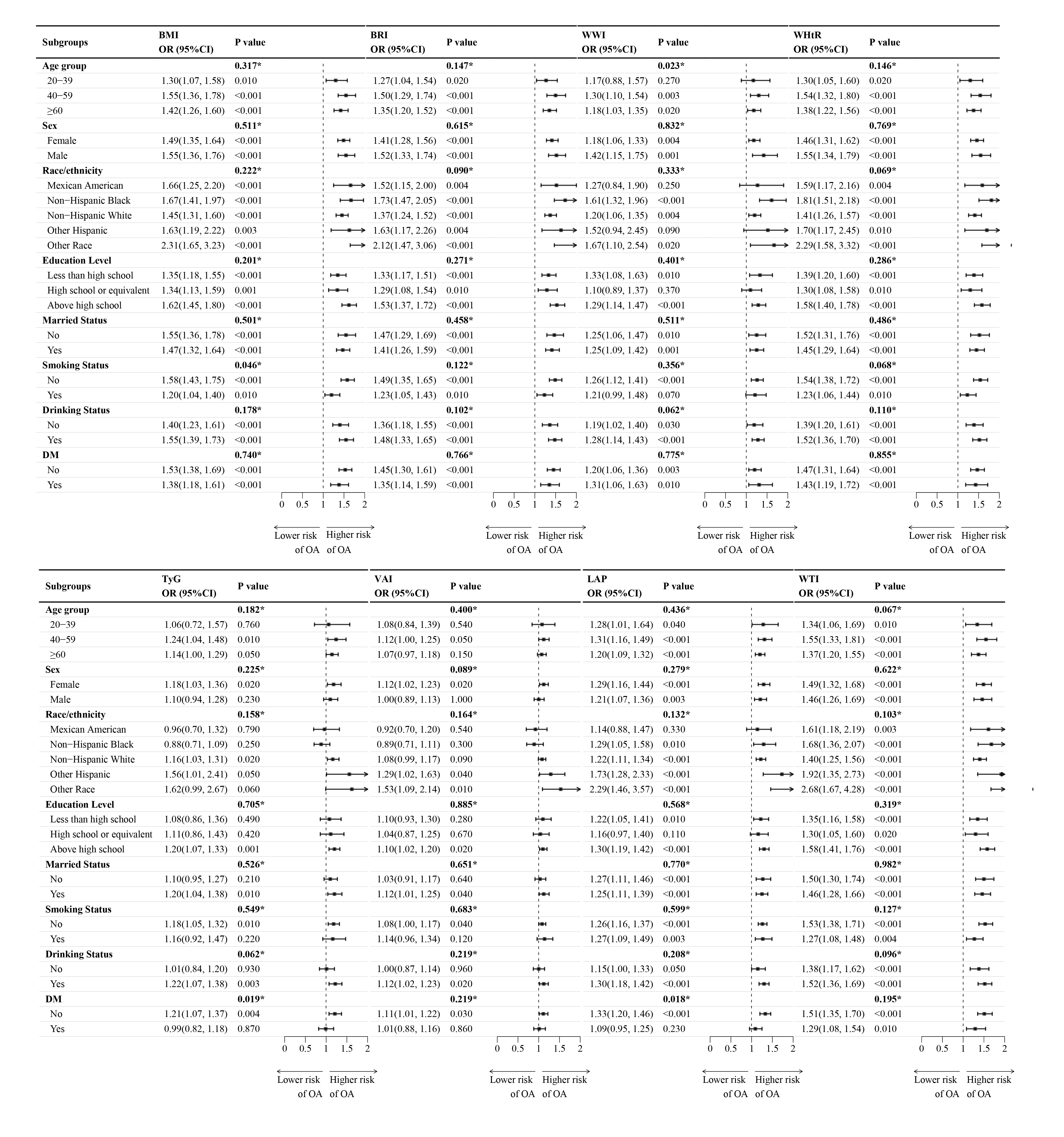
**

**Abbreviations**: BMI, body mass index; BRI, body roundness index; CI, confidence interval; DM, diabetes mellitus; FPG, fasting plasma glucose; NHANES, National Health and Nutrition Examination Survey; HbA1c, hemoglobin A1C; HDL-C, high-density lipoprotein cholesterol; LAP, lipid accumulation product; LDL-C, low-density lipoprotein cholesterol; OA, osteoarthritis; OR, odd ratio; PA, physical activity; PIR, poverty income rate; RCS, restricted cubic splines; SD, standard deviation; SE, standard error; TC, total cholesterol; TG, triglyceride; TyG, triglyceride-glucose index; VAI, visceral adiposity index; WC, waist circumference; WHtR, waist–height ratio; WTI, waist triglyceride index; WWI, weight-adjusted-waist index.

*: *P* value for interaction.

^a^. All participants aged <20 years were excluded from this analysis. And all subgroups were adjusted for age, sex, race/ethnicity, education level, marital status, smoking status, drinking status, PIR, PA time, HbA1c, TC, LDL-C, albumin level, total energy and DM.

**Supplementary Figure 2. Calibration plot showing the mean predicted probability of outcome against the observed proportion of outcomes based on the clinical OA.**





The degree of agreement between the predicted probability of the model and the actual observation probability is evaluated by the fitting degree between the drawing point and the ideal calibration line. The dashed line shows the ideal calibration line.

**Abbreviations:** BMI, body mass index; BRI, body roundness index; LAP, lipid accumulation product; OA, osteoarthritis; OR, odd ratio; PA, physical activity; TyG, triglyceride-glucose index; VAI, visceral adiposity index; WHtR, waist–height ratio; WTI, waist triglyceride index; WWI, weight-adjusted-waist index.

**Supplementary Table 1. Association of Eight anthropometric indexes with the Prevalence of Clinical Osteoarthritis in DM groups.**

|  | Crude Model^a^ | | Adjusted Model^b^ | |
| --- | --- | --- | --- | --- |
|  | OR (95%CI) | *P* value | OR (95%CI) | *P* value |
| BMI, per SD | 1.15(1.02, 1.29) | 0.02 | 1.38(1.18, 1.61) | <0.001 |
| BMI Group |  |  |  |  |
| Q1 | Reference |  | Reference |  |
| Q2 | 1.92(1.03, 3.59) | 0.04 | 2.04(1.01, 4.09) | 0.05 |
| Q3 | 2.15(1.18, 3.88) | 0.01 | 2.58(1.38, 4.83) | 0.004 |
| Q4 | 2.59(1.45, 4.62) | 0.002 | 4.02(2.11, 7.66) | <0.001 |
| *P* for trend test |  | 0.003 |  | <0.001 |
| BRI per SD | 1.28(1.13, 1.44) | <0.001 | 1.35(1.15, 1.60) | <0.001 |
| BRI, per SD |  |  |  |  |
| Q1 | Reference |  | Reference |  |
| Q2 | 1.63(0.62, 4.31) | 0.32 | 1.19(0.44, 3.18) | 0.73 |
| Q3 | 2.86(1.14, 7.17) | 0.03 | 2.21(0.88, 5.56) | 0.09 |
| Q4 | 4.33(1.73, 10.81) | 0.002 | 3.57(1.42, 9.00) | 0.01 |
| *P* for trend test |  | <0.001 |  | <0.001 |
| WWI, per SD | 1.64(1.38, 1.95) | <0.001 | 1.32(1.06, 1.64) | 0.01 |
| WWI Group |  |  |  |  |
| Q1 | Reference |  | Reference |  |
| Q2 | 1.97(0.87, 4.48) | 0.1 | 1.38(0.61, 3.14) | 0.44 |
| Q3 | 2.29(1.04, 5.04) | 0.04 | 1.53(0.72, 3.25) | 0.26 |
| Q4 | 4.43(2.13, 9.24) | <0.001 | 2.22(1.08, 4.59) | 0.03 |
| *P* for trend test |  | <0.001 |  | 0.02 |
| WHtR, per SD | 1.35(1.18, 1.54) | <0.001 | 1.43(1.19, 1.73) | <0.001 |
| WHtR Group |  |  |  |  |
| Q1 | Reference |  | Reference |  |
| Q2 | 1.63(0.62, 4.31) | 0.32 | 1.19(0.44, 3.18) | 0.73 |
| Q3 | 2.86(1.14, 7.17) | 0.03 | 2.21(0.88, 5.56) | 0.09 |
| Q4 | 4.33(1.73, 10.81) | 0.002 | 3.57(1.42, 9.00) | 0.01 |
| *P* for trend test |  | <0.001 |  | <0.001 |
| TyG, per SD | 0.95(0.83, 1.09) | 0.5 | 0.99(0.83, 1.19) | 0.94 |
| TyG Group |  |  |  |  |
| Q1 | Reference |  | Reference |  |
| Q2 | 0.64(0.33, 1.25) | 0.19 | 0.56(0.28, 1.13) | 0.1 |
| Q3 | 1.05(0.60, 1.83) | 0.86 | 0.98(0.56, 1.74) | 0.95 |
| Q4 | 0.74(0.45, 1.20) | 0.21 | 0.68(0.39, 1.18) | 0.17 |
| *P* for trend test |  | 0.34 |  | 0.47 |
| LAP, per SD | 1.08(0.96, 1.21) | 0.18 | 1.09(0.95, 1.26) | 0.21 |
| LAP Group |  |  |  |  |
| Q1 | Reference |  | Reference |  |
| Q2 | 0.92(0.43, 1.94) | 0.82 | 0.74(0.36, 1.51) | 0.4 |
| Q3 | 1.49(0.78, 2.86) | 0.23 | 1.29(0.70, 2.39) | 0.4 |
| Q4 | 1.55(0.78, 3.08) | 0.21 | 1.44(0.71, 2.92) | 0.31 |
| *P* for trend test |  | 0.03 |  | 0.04 |
| VAI, per SD | 1.07(0.94, 1.22) | 0.32 | 1.01(0.88, 1.17) | 0.84 |
| VAI Group |  |  |  |  |
| Q1 | Reference |  | Reference |  |
| Q2 | 1.08(0.66, 1.77) | 0.75 | 0.95(0.57, 1.59) | 0.84 |
| Q3 | 1.15(0.77, 1.71) | 0.5 | 1.01(0.63, 1.59) | 0.98 |
| Q4 | 1.13(0.75, 1.72) | 0.55 | 1.01(0.62, 1.67) | 0.96 |
| *P* for trend test |  | 0.59 |  | 0.86 |
| WTI, per SD | 1.10(0.96, 1.25) | 0.18 | 1.30(1.08, 1.56) | 0.01 |
| WTI Group |  |  |  |  |
| Q1 | Reference |  | Reference |  |
| Q2 | 0.97(0.41, 2.32) | 0.94 | 0.73(0.29, 1.87) | 0.51 |
| Q3 | 1.40(0.64, 3.07) | 0.4 | 1.38(0.58, 3.27) | 0.46 |
| Q4 | 1.64(0.77, 3.49) | 0.2 | 1.94(0.81, 4.65) | 0.14 |
| *P* for trend test |  | 0.02 |  | <0.001 |

**Abbreviations:** BMI, body mass index; BRI, body roundness index; CI, confidence interval; DM, diabetes mellitus; FPG, fasting plasma glucose; NHANES, National Health and Nutrition Examination Survey; HbA1c, hemoglobin A1C; HDL-C, high-density lipoprotein cholesterol; LAP, lipid accumulation product; LDL-C, low-density lipoprotein cholesterol; OA, osteoarthritis; OR, odd ratio; PA, physical activity; PIR, poverty income rate; SD, standard deviation; SE, standard error; TC, total cholesterol; TG, triglyceride; TyG, triglyceride-glucose index; VAI, visceral adiposity index; WC, waist circumference; WHtR, waist–height ratio; WTI, waist triglyceride index; WWI, weight-adjusted-waist index.

^a^. Crude model: No adjustment for confounding factors.

^b^. Adjusted for age, sex, race/ethnicity, education level, marital status, smoking status, drinking status, PIR, PA time, HbA1c, TC, LDL-C, albumin level, and total energy.

**Supplementary Table 2. The AUC and thresholds of weighted ROC curves for comparing all indexes.**

|  | **AUC (95%CI)** | **Thresholds**  **(Specificity and sensitivity)** | **AIC** | **BIC** |
| --- | --- | --- | --- | --- |
| WWI | 0.6724 (0.66,0.69) | 11.124 (0.591,0.675) | 8586.9 | 8611.8 |
| WHtR | 0.6448 (0.63,0.66) | 0.595 (0.585, 0.626) | 8780.8 | 8804.9 |
| BRI | 0.6448 (0.63,0.66) | 5.322 (0.585.0.626) | 8811.4 | 8835.3 |
| WTI | 0.6159 (0.60,0.63) | 853.345 (0.550,0.616) | 8882.4 | 8906.6 |
| LAP | 0.6096 (0.59,0.62) | 38.613 (0.489,0.680) | 8940.2 | 8964.9 |
| BMI | 0.5906 (0.57,0.61) | 30.795 (0.690, 0.443) | 8959.5 | 8983.3 |
| VAI | 0.5567 (0.54,0.57) | 1.452 (0.538,0.553) | 9034.4 | 9058.9 |
| TyG | 0.5695 (0.55,0.58) | 8.547 (0.510,0.607) | 8981.7 | 9006.3 |

**Abbreviations:** AIC, Akaike Information Criterion; AUC, area under curve; BMI, body mass index; BIC, Bayesian Information Criterion; BRI, body roundness index; CI, confidence interval; LAP, lipid accumulation product; ROC, receiver operating characteristic; TyG, triglyceride-glucose index; VAI, visceral adiposity index; WHtR, waist–height ratio; WTI, waist triglyceride index; WWI, weight-adjusted-waist index.

**Supplementary Table 3. Association of Eight anthropometric indexes with the Prevalence of Clinical Osteoarthritis after excluding the upper and lower 1% extremes.**

|  | Crude Model^a^ | | Adjusted Model^b^ | |
| --- | --- | --- | --- | --- |
|  | OR (95%CI) | *P* value | OR (95%CI) | *P* value |
| BMI, per SD | 1.29(1.19, 1.41) | <0.001 | 1.48(1.34, 1.64) | <0.001 |
| BMI Group |  |  |  |  |
| Q1 | Reference |  | Reference |  |
| Q2 | 1.53(1.19, 1.96) | <0.001 | 1.62(1.22, 2.15) | 0.001 |
| Q3 | 1.40(1.09, 1.79) | 0.01 | 1.67(1.24, 2.24) | <0.001 |
| Q4 | 2.11(1.64, 2.72) | <0.001 | 2.94(2.19, 3.94) | <0.001 |
| *P* for trend |  | <0.001 |  | <0.001 |
| BRI, per SD | 1.49(1.38, 1.62) | <0.001 | 1.42(1.28, 1.58) | <0.001 |
| BRI Group |  |  |  |  |
| Q1 | Reference |  | Reference |  |
| Q2 | 1.67(1.30, 2.14) | <0.001 | 1.29(0.97, 1.72) | 0.08 |
| Q3 | 2.18(1.70, 2.79) | <0.001 | 1.64(1.21, 2.22) | 0.002 |
| Q4 | 3.43(2.66, 4.43) | <0.001 | 2.56(1.88, 3.50) | <0.001 |
| *P* for trend |  | <0.001 |  | <0.001 |
| WWI, per SD | 1.82(1.67, 1.98) | <0.001 | 1.23(1.09, 1.38) | 0.001 |
| WWI Group |  |  |  |  |
| Q1 | Reference |  | Reference |  |
| Q2 | 2.02(1.55, 2.64) | <0.001 | 1.35(1.02, 1.78) | 0.03 |
| Q3 | 2.90(2.29, 3.68) | <0.001 | 1.42(1.08, 1.86) | 0.01 |
| Q4 | 4.66(3.63, 5.99) | <0.001 | 1.53(1.10, 2.13) | 0.01 |
| *P* for trend |  | <0.001 |  | 0.03 |
| WHtR, per SD | 1.53(1.41, 1.67) | <0.001 | 1.45(1.30, 1.62) | <0.001 |
| WHtR Group |  |  |  |  |
| Q1 | Reference |  | Reference |  |
| Q2 | 1.67(1.30, 2.14) | <0.001 | 1.29(0.97, 1.72) | 0.08 |
| Q3 | 2.18(1.70, 2.79) | <0.001 | 1.64(1.21, 2.22) | 0.002 |
| Q4 | 3.43(2.66, 4.43) | <0.001 | 2.56(1.88, 3.50) | <0.001 |
| *P* for trend |  | <0.001 |  | <0.001 |
| TyG, per SD | 1.29(1.18, 1.41) | <0.001 | 1.18(1.04, 1.32) | 0.01 |
| TyG Group |  |  |  |  |
| Q1 | Reference |  | Reference |  |
| Q2 | 1.09(0.85, 1.39) | 0.48 | 0.90(0.69, 1.19) | 0.47 |
| Q3 | 1.59(1.26, 2.02) | <0.001 | 1.31(1.01, 1.72) | 0.05 |
| Q4 | 1.89(1.45, 2.46) | <0.001 | 1.42(1.02, 1.98) | 0.04 |
| *P* for trend |  | <0.001 |  | 0.01 |
| LAP, per SD | 1.32(1.22, 1.43) | <0.001 | 1.28(1.17, 1.41) | <0.001 |
| LAP Group |  |  |  |  |
| Q1 | Reference |  | Reference |  |
| Q2 | 1.55(1.21, 1.97) | <0.001 | 1.31(0.99, 1.73) | 0.05 |
| Q3 | 2.26(1.76, 2.92) | <0.001 | 1.91(1.40, 2.60) | <0.001 |
| Q4 | 2.66(2.03, 3.48) | <0.001 | 2.17(1.59, 2.95) | <0.001 |
| *P* for trend |  | <0.001 |  | <0.001 |
| VAI, per SD | 1.15(1.07, 1.25) | <0.001 | 1.11(1.01, 1.22) | 0.04 |
| VAI Group |  |  |  |  |
| Q1 | Reference |  | Reference |  |
| Q2 | 1.16(0.91, 1.47) | 0.22 | 1.17(0.89, 1.54) | 0.25 |
| Q3 | 1.45(1.17, 1.78) | <0.001 | 1.34(1.05, 1.73) | 0.02 |
| Q4 | 1.62(1.27, 2.07) | <0.001 | 1.46(1.10, 1.95) | 0.01 |
| *P* for trend |  | <0.001 |  | 0.01 |
| WTI, per SD | 1.41(1.29, 1.54) | <0.001 | 1.46(1.30, 1.63) | <0.001 |
| WTI Group |  |  |  |  |
| Q1 | Reference |  | Reference |  |
| Q2 | 1.66(1.30, 2.10) | <0.001 | 1.52(1.16, 2.00) | 0.003 |
| Q3 | 1.92(1.50, 2.45) | <0.001 | 1.84(1.34, 2.52) | <0.001 |
| Q4 | 2.68(2.11, 3.40) | <0.001 | 2.65(1.98, 3.53) | <0.001 |
| *P* for trend |  | <0.001 |  |  |

**Abbreviations:** BMI, body mass index; BRI, body roundness index; CI, confidence interval; DM, diabetes mellitus; FPG, fasting plasma glucose; NHANES, National Health and Nutrition Examination Survey; HbA1c, hemoglobin A1C; HDL-C, high-density lipoprotein cholesterol; LAP, lipid accumulation product; LDL-C, low-density lipoprotein cholesterol; OA, osteoarthritis; OR, odd ratio; PA, physical activity; PIR, poverty income rate; SD, standard deviation; SE, standard error; TC, total cholesterol; TG, triglyceride; TyG, triglyceride-glucose index; VAI, visceral adiposity index; WC, waist circumference; WHtR, waist–height ratio; WTI, waist triglyceride index; WWI, weight-adjusted-waist index.

^a^. Crude model: No adjustment for confounding factors.

^b^. Adjusted for age, sex, race/ethnicity, education level, marital status, smoking status, drinking status, PIR, PA time, HbA1c, TC, LDL-C, albumin level, total energy and DM.

**Supplementary Table 4. Association of Eight anthropometric indexes with the Prevalence of Clinical Osteoarthritis after excluding those who have diabetes or long-term use of hypoglycemic and lipid-lowering drugs for sensitivity analyses.**

|  | Crude Model^a^ | | Adjusted Model^b^ | |
| --- | --- | --- | --- | --- |
|  | OR (95%CI) | *P* value | OR (95%CI) | *P* value |
| BMI, per SD | 1.30(1.19, 1.42) | <0.001 | 1.49(1.35, 1.65) | <0.001 |
| BMI Group |  |  |  |  |
| Q1 | Reference |  | Reference |  |
| Q2 | 1.39(1.04, 1.85) | 0.02 | 1.37(1.00, 1.88) | 0.05 |
| Q3 | 1.35(0.99, 1.84) | 0.06 | 1.55(1.08, 2.21) | 0.02 |
| Q4 | 1.90(1.46, 2.48) | <0.001 | 2.46(1.83, 3.30) | <0.001 |
| *P* for trend |  | <0.001 |  | <0.001 |
| BRI, per SD | 1.44(1.32, 1.57) | <0.001 | 1.39(1.25, 1.54) | <0.001 |
| BRI Group |  |  |  |  |
| Q1 | Reference |  | Reference |  |
| Q2 | 1.81(1.30, 2.50) | <0.001 | 1.40(0.96, 2.02) | 0.08 |
| Q3 | 2.38(1.80, 3.15) | <0.001 | 1.67(1.17, 2.36) | 0.005 |
| Q4 | 3.47(2.60, 4.65) | <0.001 | 2.49(1.78, 3.49) | <0.001 |
| *P* for trend |  | <0.001 |  | <0.001 |
| WWI, per SD | 1.75(1.59, 1.93) | <0.001 | 1.14(1.01, 1.29) | 0.04 |
| WWI Group |  |  |  |  |
| Q1 | Reference |  | Reference |  |
| Q2 | 1.92(1.33, 2.75) | <0.001 | 1.23(0.87, 1.75) | 0.24 |
| Q3 | 3.67(2.72, 4.95) | <0.001 | 1.84(1.33, 2.54) | <0.001 |
| Q4 | 4.83(3.53, 6.62) | <0.001 | 1.53(1.06, 2.22) | 0.02 |
| *P* for trend |  | <0.001 |  | 0.01 |
| WHtR, per SD | 1.49(1.37, 1.63) | <0.001 | 1.41(1.26, 1.58) | <0.001 |
| WHtR Group |  |  |  |  |
| Q1 | Reference |  | Reference |  |
| Q2 | 1.81(1.30, 2.50) | <0.001 | 1.40(0.96, 2.02) | 0.08 |
| Q3 | 2.38(1.80, 3.15) | <0.001 | 1.67(1.17, 2.36) | 0.005 |
| Q4 | 3.47(2.60, 4.65) | <0.001 | 2.49(1.78, 3.49) | <0.001 |
| *P* for trend |  | <0.001 |  | <0.001 |
| TyG, per SD | 1.27(1.16, 1.38) | <0.001 | 1.15(1.01, 1.29) | 0.03 |
| TyG Group |  |  |  |  |
| Q1 | Reference |  | Reference |  |
| Q2 | 1.29(0.94, 1.79) | 0.12 | 0.95(0.67, 1.34) | 0.78 |
| Q3 | 1.68(1.18, 2.39) | 0.004 | 1.21(0.85, 1.70) | 0.28 |
| Q4 | 1.76(1.30, 2.38) | <0.001 | 1.19(0.84, 1.69) | 0.32 |
| *P* for trend |  | <0.001 |  | 0.16 |
| LAP, per SD | 1.31(1.22, 1.40) | <0.001 | 1.27(1.15, 1.40) | <0.001 |
| LAP Group |  |  |  |  |
| Q1 | Reference |  | Reference |  |
| Q2 | 2.33(1.62, 3.35) | <0.001 | 1.87(1.28, 2.74) | 0.002 |
| Q3 | 2.71(1.90, 3.86) | <0.001 | 1.97(1.32, 2.94) | 0.001 |
| Q4 | 3.01(2.24, 4.05) | <0.001 | 2.20(1.56, 3.08) | <0.001 |
| *P* for trend |  | <0.001 |  | <0.001 |
| VAI, per SD | 1.17(1.08, 1.26) | <0.001 | 1.10(0.99, 1.22) | 0.08 |
| VAI Group |  |  |  |  |
| Q1 | Reference |  | Reference |  |
| Q2 | 1.25(0.94, 1.65) | 0.13 | 1.13(0.82, 1.55) | 0.44 |
| Q3 | 1.33(0.96, 1.84) | 0.09 | 1.22(0.86, 1.73) | 0.26 |
| Q4 | 1.59(1.21, 2.09) | 0.001 | 1.30(0.93, 1.80) | 0.12 |
| *P* for trend |  | 0.001 |  | 0.12 |
| WTI, per SD | 1.39(1.27, 1.52) | <0.001 | 1.43(1.28, 1.59) | <0.001 |
| WTI Group |  |  |  |  |
| Q1 | Reference |  | Reference |  |
| Q2 | 1.82(1.34, 2.49) | <0.001 | 1.59(1.12, 2.27) | 0.01 |
| Q3 | 2.13(1.58, 2.87) | <0.001 | 1.85(1.26, 2.70) | 0.002 |
| Q4 | 2.56(1.92, 3.41) | <0.001 | 2.35(1.66, 3.33) | <0.001 |
| *P* for trend |  | <0.001 |  | <0.001 |

**Abbreviations:** BMI, body mass index; BRI, body roundness index; CI, confidence interval; DM, diabetes mellitus; FPG, fasting plasma glucose; NHANES, National Health and Nutrition Examination Survey; HbA1c, hemoglobin A1C; HDL-C, high-density lipoprotein cholesterol; LAP, lipid accumulation product; LDL-C, low-density lipoprotein cholesterol; OA, osteoarthritis; OR, odd ratio; PA, physical activity; PIR, poverty income rate; SD, standard deviation; SE, standard error; TC, total cholesterol; TG, triglyceride; TyG, triglyceride-glucose index; VAI, visceral adiposity index; WC, waist circumference; WHtR, waist–height ratio; WTI, waist triglyceride index; WWI, weight-adjusted-waist index.

^a^. Crude model: No adjustment for confounding factors.

^b^. Adjusted for age, sex, race/ethnicity, education level, marital status, smoking status, drinking status, PIR, PA time, HbA1c, TC, LDL-C, albumin level and total energy.
